# Contusion Progression Following Traumatic Brain Injury: A Review of Clinical and Radiological Predictors, and Influence on Outcome

**DOI:** 10.1007/s12028-020-00994-4

**Published:** 2020-05-27

**Authors:** Krishma Adatia, Virginia F. J. Newcombe, David K. Menon

**Affiliations:** Division of Anaesthesia, University of Cambridge, Cambridge, UK

**Keywords:** Contusion, Progression, Predictors, Traumatic brain injury, Outcome

## Abstract

Secondary injuries remain an important cause of the morbidity and mortality associated with traumatic brain injury (TBI). Progression of cerebral contusions occurs in up to 75% of patients with TBI, and this contributes to subsequent clinical deterioration and requirement for surgical intervention. Despite this, the role of early clinical and radiological factors in predicting contusion progression remains relatively poorly defined due to studies investigating progression of all types of hemorrhagic injuries as a combined cohort. In this review, we summarize data from recent studies on factors which predict contusion progression, and the effect of contusion progression on clinical outcomes.

## Introduction

Traumatic brain injury (TBI) is a heterogeneous disease, encompassing a spectrum of pathological features from axonal to hemorrhagic injuries. Of these, cerebral contusions are a significant contributor to death and disability following TBI and occur in up to 35% of severe cases [1]. Exacerbation of these injuries often occurs as a result of ongoing pathophysiological mechanisms initiated at the time of primary injury; progression of traumatic contusions, in particular, is an important secondary injury which contributes to subsequent clinical deterioration and requirement for surgical intervention [2]. Among the various subtypes of intracranial hemorrhage, contusions are most likely to progress [3–5]; in the majority of patients, this occurs within the first 24 h [6–9], with very few progressing after 3–4 days [7, 10].

A reported 16–75% of contusions show progression in subsequent imaging [2, 6–9, 11–23]. This disparity in reported percentages is, in part, due to a lack of standardized definition of contusion progression across the literature. The threshold above which an increase in contusion size is defined as progression is a major source of this discrepancy. Among studies using relative volume changes, for example, the definition of progression ranges from a 5% increase in volume in one study to a 50% increase in volume in another [2, 6, 8, 11, 13, 14, 17, 18, 23]. Similarly, in studies using absolute volume changes, this definition varies between a 1 cm increase in diameter and a 12.5 ml volume increase [7, 9, 12, 15]. One study has used a combination of absolute and relative thresholds [22]. Different scan intervals and study inclusion criteria also contribute to this variation in reported percentages. Methods to assess contusion volume include the ABC/2 formula, and manual or automated image segmentation. Differences in the accuracy of volume assessment between these methods may additionally contribute to this discrepancy; however, the majority of studies use the ABC/2 method, and large variations in incidence of progression exist even among these studies.

Given the high rate and early time course of this phenomenon, identification of factors predictive of contusion progression is important in order to stratify TBI patients and tailor initial clinical management; in those at high risk of progression, early surgical intervention may be beneficial, for example. However, whether progression represents an inevitable stage in the natural history of traumatic contusions, or a secondary injury which may be prevented, is unclear.

Despite the higher likelihood of contusion progression compared to other lesions, many studies investigate the phenomenon of ‘progressive haemorrhagic injury,’ which represents progression of subarachnoid hemorrhages (SAH), subdural hemorrhages (SDH), and extradural hemorrhages (EDH), in addition to contusions [3, 5, 24–27]; this has resulted in limited appreciation of the risk factors that are specific to the progression of contusions.

In this review, we discuss the phenomenon of contusion progression, factors associated with progression, and its influence on clinical outcomes; a summary of the studies discussed is shown in Table 1. Given computed tomography (CT) is the most common imaging modality used in the acute stage of TBI to monitor progression, and the current literature is almost entirely based on the hemorrhagic component of progression, this review focuses on these aspects.

**Table 1:** Summary of studies investigating factors predictive of contusion progression and effect of progression on outcomes

Study	Year	Design	Number of patients	Injury severity	Method to measure contusion volume	Definition of progression	% showing progression	Timing of CT scans	Independent predictors of progression	Effect of progression on outcome
Rehman et al.	2019	Prospective	246	Any	ABC/2	>30%	44.7%	Data not given	Frontal contusion Bilateral contusions Initial contusion volume > 20 ml Multiple contusions Presence of SDH Presence of SAH	No difference in in-hospital mortality rate
Carnevale et al.	2018	Retrospective	491	Any	3D imaging software	Any increase	74.7%	Two scans within 72 h of injury	Univariate analysis only: Age ISS and NISS GCS Absolute platelet count Presence of SDH	Association with discharge disposition, i.e., home, skilled nursing facility, or hospice/death in univariate analysis
Wan et al.	2017	Retrospective	181	Any	ABC/2	≥ 33%	37.6%	Within 6 h of injury then at 12, 24, and 72 h after admission	History of hypertension Linear bone fracture INR > 1.2	No difference in mortality rate or unfavorable outcome (GOS ≤ 3) at 6 months Association with requirement for delayed operation in univariate analysis
Sharma et al.	2016	Prospective	110	Any	ABC/2	>30%	45.45%	Two scans within 72 h after injury	Coagulopathy Presence of SDH Presence of SAH	Association between change in contusion volume and surgical intervention in univariate but not multivariate analysis
Cepeda et al.	2016	Retrospective	408	Moderate and severe	Volumetric software	≥ 33% or new lesion	65.9%	Two scans within 72 h after injury	Initial contusion volume < 1 ml Cisternal compression Decompressive craniectomy Falls Multiple TICH Contrecoup TICH Presence of SDH	Association between progression and unfavorable outcome (GOS ≤ 3) at 6 months Association between change in contusion volume and 6-month outcome
Allison et al.	2016	Retrospective	286	Moderate and severe	ABC/2	≥ 30% and ≥ 10 ml	21%	Two scans within 24 h of injury	Presence of SDH Presence of SAH Presence of skull fracture RBC transfusion	
Cepeda et al.	2015	Retrospective	782	Moderate and severe	ABC/2	≥ 33% or new lesion	64%	Second scan–worst CT during admission, mean time 30.1 h from trauma	Initial volume < 5 ml Cisternal compression Decompressive craniectomy Older age Falls Multiple TICH Hypoxia	
Qureshi et al.	2015	Prospective	1200	Severe	–	Any increase in size	19.8%	First 3 CTs within the first week of injury	Univariate analysis only: Initial systolic blood pressure and systolic blood pressure > 140 ISS and ISS > 26 Initial Marshall score Admission GCS ≤ 5 Serum sodium > 145 mEq/L at 12–24 h	Univariate associations only: In-hospital adverse events: ≥ 1 nosocomial infection, pneumonia, bloodstream infection, urinary tract infection, wound infection Requirement for ventriculostomy or craniotomy in first 24 h or 5 days 6-month GOSE ≤ 4 1-month and 6-month DRS 28-day and 6-month survival Ventilator free days Total ICU days Total hospital days Multivariate association: 6-month GOSE ≤ 4
Kim et al.	2015	Retrospective	56	Mild and moderate	ABC/2	≥ 30%	55%	Repeat scans at 4 and 24 h following initial scan	Smoking Triglyceride level < 150 mg/dL	
Iaccarino et al.	2014	Retrospective	352	Any	ABC/2	30%	65.5% between first and second 36.8% between second and third overall 42.3%	Mean time to second scan 9 h, mean time to third scan 38 h	Initial contusion volume	Association with 6-month GOSE ≤ 4 in univariate analysis, but not multivariate Association with clinical deterioration in univariate analysis, but not multivariate
Juratli et al.	2014	Prospective	153	Any	–	≥ 1 cm increase in diameter, or new lesion > 1 cm	43.5%	Admission and 6 h later	Platelet count < 100 × 10^9^/L	No association with late surgery, in-hospital mortality, or hospital length of stay Univariate associations: Discharge mRS ≥ 4 12-month mRS ≥ 4 Ventilation hours ICU length of stay Multivariate associations: mRS ≥ 4 at discharge and 12 months
Alahmadi et al.	2010	Retrospective	98	Any	ABC/2	≥ 30%	45%	Two scans during hospital admission	Volume of contusion on admission Presence of SDH	Univariate association with need for neurosurgical intervention, excluding tracheostomy No association with patient disposition
White et al.	2009	Retrospective	46	Any	ABC/2	≥ 33%	65%	Two scans within 24 h of injury	Univariate analysis only: INR and INR > 1.2 Admission GCS GCS at time of scan 1 GCS at time of scan 2	Univariate association with discharge disposition Association with in-hospital mortality after adjusting for ISS only
Narayan et al.	2008	Prospective	56	GCS 4–14	ABC/2	Any increase in volume	51% at 24 h 53% at 72 h	Admission, and 24 and 72 h after injury	Univariate analysis only: Initial contusion size	No association between change in lesion volume and Barthel Index at discharge or day 15
Chang et al.	2006	Retrospective	113	Any	ABC/2	Any increase in volume	35% between first and second scan 38% between first and last scan	Two or more scans within 72 h of admission	Presence of SAH Presence of SDH Initial contusion size For increase > 5 cm^3^ Presence of SDH Initial contusion size	Growth of hematoma volume > 5 cm^3^ independently associated with surgery
Yadav et al.	2006	Prospective	262	Any	ABC/2	12.5 ml	16.4%	Two scans within 24 h of admission	Univariate analysis only: GCS Midline shift Coagulopathy	
Beaumont and Gennarelli	2006	Retrospective	21	Any	Manual segmentation	> 5%	47.6%	Two scans within 24 h of admission	Univariate analysis only: Ratio of edema: no edema	

DRS, Disability Rating Scale score; GCS, Glasgow Coma Score; GOS, Glasgow Outcome Score; GOSE, Extended Glasgow Outcome Score; ICU, intensive care unit; ISS, injury severity score; mRS, modified Rankin Scale score; NISS, new injury severity score; RBC, red blood cell; SAH, subarachnoid hemorrhage; SDH, subdural hemorrhage; TICH, traumatic intracerebral hemorrhage

## Pathophysiology of contusion progression

Contusion progression has historically been attributed to continued bleeding from fractured microvessels, exacerbated by coagulopathy [28]. In recent years, however, the idea of a ‘traumatic penumbra’ surrounding the contusion core that is metabolically compromised and thus more vulnerable to secondary insults has been proposed [10, 29, 30]. In such areas, a reduction in cerebral blood flow (CBF) may precede contusion expansion [31–35]. Indeed, on magnetic resonance imaging (MRI) performed within the first 72 days of injury, a cytotoxic rim of edema is seen on diffusion tensor imaging that is subsumed by vasogenic edema as the lesion progresses [30]. Kurland et al. have suggested the role of microvascular dysfunction within this penumbra; they propose that, in the region that will become the contusion core, the kinetic energy received during impact results in the fracturing of microvessels. Although the surrounding penumbra also receives kinetic energy, this is insufficient to fracture microvessels, instead activating two transcription factors: specificity protein 1 and nuclear factor-ĸB [10]. These subsequently lead to the upregulation of sulfonylurea receptor 1 which increases blood–brain barrier (BBB) permeability and augments the formation of ionic and vasogenic edema [36, 37]. Oncotic death of endothelial cells and capillary fragmentation follows, resulting in extravasation of blood from capillaries, at which point the contusion is said to progress.

## Clinical predictors

1. Baseline demographics

Given the proposed mechanisms described above, patient demographics may provide insights into the likelihood of contusion progression: Preexisting vessel fragility may predispose some patients to ongoing secondary damage.

Although some studies have shown associations between increasing patient age [6, 11, 14, 20, 22] or male gender [3, 11] and contusion progression in univariate analyses, neither are independent predictors for progression. In older patients, it is thought that age-associated structural weaknesses in microvasculature, loss of endothelium, and reduced resting CBF contribute to greater vulnerability to the mechanisms involved in progression within the traumatic penumbra [38, 39]. Meanwhile, in female patients, estrogen and progesterone may be neuroprotective; the mechanisms by which this neuroprotection may occur include greater membrane stability due to reduced lipid peroxidation [40], reduced apoptosis via bcl-2 upregulation [41], and increased CBF during periods of ischemia [42].

In a study by Wan et al., past medical history of hypertension was an independent predictor for contusion progression, with such patients being 4.5 times more likely to show progression than those who were not known to be hypertensive [21]. Chronic hypertension induces cerebrovascular remodeling and endothelial dysfunction, resulting in an intrinsic increased BBB permeability when compared to normotensive patients [43–47]. Since increased BBB permeability is an important stage in the development of contusion progression, the higher baseline BBB permeability among hypertensive patients may result in an inherently higher risk of developing cerebral edema, and subsequent contusion progression. Chronic hypertension also causes a rightward shift of the cerebral autoregulation curve, resulting in an increase in the lower limit of autoregulation [46]. Hypertensive patients may thus be more susceptible to the reductions in CBF seen in the penumbral region.

Current cigarette smoking increases risk of contusion progression by sixfold [18]. In a similar manner to the mechanisms described above, current cigarette smoking is associated with greater vessel fragility as well as reduced CBF [47]; in smokers with a background chronic reduction in CBF, the additional contribution of reduced CBF within the penumbral zone may increase the likelihood of progression.

2. Injury characteristics

The Glasgow Coma Score (GCS) is an indicator of injury severity, and initial GCS has been identified as a predictor of contusion progression [7, 17, 20]. White et al. showed that those with an initial GCS ≥ 14 were less likely to experience subsequent progression compared to those with GCS < 14 [8], and in a study by Qureshi et al., presence of progression was associated with an initial GCS ≤ 5 [16]. In addition to a greater incidence of progression, Carnevale et al. reported that those with a lower GCS on admission (≤ 8) had a greater rate of progression than those with GCS > 8 [20]. Associations between initial GCS and contusion progression have not been seen in all studies [2, 3, 9, 11, 12, 14], though this may be attributable to unreliable GCS scores during pre-hospital care or due to the interference of sedation with neurological assessment of patients on admission; the clinical utility of the initial GCS score for the prediction of contusion progression may therefore be limited.

The mechanism of trauma may also be important in determining whether contusion progression is likely or not, though this has not been studied in great detail. Cepeda et al. have reported progression to occur more frequently among those who sustain TBI as a result of a fall compared to a road traffic accident [14, 23]. Since TBI secondary to a fall is more common among older age groups and alcoholic patients, this association may be reflective of patient demographics and baseline coagulation status rather than mechanism of injury itself.

3. Admission blood pressure

Although a history of hypertensive disease may contribute to contusion progression, as discussed above, only two studies have suggested some association between higher admission systolic blood pressure and progression [16, 18]. The apparent lack of association between admission blood pressure and progression seen in most studies [3, 12, 20, 21, 26] may be a result of hypotensive patients receiving aggressive fluid resuscitation, which itself may contribute to contusion progression. It may also be that blood pressure during the course of admission is more important than a single measurement.

4. Laboratory parameters

Coagulopathy is a common finding in TBI, with up to 63% of patients with severe TBI displaying abnormal coagulation tests on admission [7, 8, 15]. The predictive value of admission coagulopathy on lesion progression has predominantly been studied in the context of progressive hemorrhagic injury, with few studies examining its role in predicting progression of contusions specifically. Despite its common occurrence, the reliability of coagulopathy in predicting contusion progression therefore remains controversial. This is also likely due to varying definitions of coagulopathy between studies, early administration of coagulation products, and potential exclusion of patients with an initial coagulopathy due to emergent surgical evacuation; Allison et al. have reported a reduced risk of progression in patients who receive early red blood cell transfusion [22].

Many studies on contusion progression report no association with coagulopathy [12, 14, 16, 22]. Among those in which coagulopathy does predict progression, INR and platelet count appear to be of particular importance. For each unit increase in INR, risk of contusion progression increases sevenfold [6], with patients who exhibit an INR > 1.2 being almost three times more likely to experience progression than those with an INR ≤ 1.2 [8, 21]. In a study by Juratli et al., a platelet count < 100 × 10^9^/L was associated with a close to sixfold increased risk of progression [15]. History of anticoagulation or anti-platelet use does not appear to be associated with progression [2].

Low triglyceride levels (< 150 mg/dL) are associated with a fourfold increased risk of contusion progression [18]. Since triglycerides contribute to overall cholesterol levels, an important constituent of cell membranes, patients with low triglyceride levels may have more fragile endothelium which is more prone to fracturing following TBI [48]. Additionally, studies have shown higher frequency of cerebral microbleeds in patients with low triglyceride levels [49].

Alcohol intoxication is known to impair platelet function and contribute to coagulopathy [50]. Although Carnevale et al. found blood alcohol level to correlate with contusion size on follow-up scan [20], they, along with several other studies [3, 15], did not find this to be predictive of presence of progression. In addition to its anti-platelet effects, alcohol reduces vascular tone [51]; systemic hypotension may therefore counteract the anti-platelet effects of alcohol on contusion progression.

## Radiological predictors

Radiological investigations in the acute phase of brain injury, particularly CT scans, are vital in the assessment of TBI patients. The initial CT performed at admission provides information regarding the type and extent of intracranial pathologies present and determines the need for emergent neurosurgical intervention. Since such imaging is widely performed, identification of early radiological factors that favor the progression of traumatic contusions would be of great clinical use. Timing of the initial scan, however, also appears to be associated with contusion progression, with patients who have scans performed closer to the time of injury being more likely to show progression on subsequent imaging than those with a longer interval between injury and first scan [9, 52].

1. Baseline contusion characteristics

Initial contusion volume is the most widely described baseline CT characteristic to be associated with contusion progression [2, 9, 12, 17, 20]; larger initial contusions are more likely to progress, with each additional cubic centimeter volume conferring an additive risk of 11% [12]. Iaccarino et al. reported that contusions with an initial volume ≤ 4 ml are unlikely to progress, and in their study, this cutoff had a sensitivity of 95% and specificity of 75% for predicting the absence of progression [13]. For contusions initially larger than 20 ml, risk of progression is increased by fivefold [11]. As well as an increased likelihood of progression, larger initial contusions demonstrate a greater degree of progression [8, 12, 20]. Although studies consistently demonstrate that larger lesions are more likely to progress, Cepeda et al. found the converse, suggesting that small lesions may have more space in which to expand, compared to larger lesions which need to overcome higher pressures in order to increase in volume [14, 23].

Contusions associated with TBI are most commonly seen in frontal and temporal lobes as a result of impact with bone, but may be seen throughout the brain [7, 12, 13]. Studies have suggested contusion location to be an important predictor of subsequent growth, with frontal contusions being 1.5 times more likely to progress as compared to other locations [11, 22]. Contrecoup contusions, defined as those located more than 90° from site of impact, are almost twice as likely to progress compared to coup contusions [23]. Additionally, among contrecoup contusions, those within the temporal lobe are more likely to progress compared to those in frontal or posterior regions [23]. Patients with bilateral or multiple contusions are also threefold more likely to experience contusion progression than those with either unilateral or single contusions [11, 14]. This association may represent a more severe underlying initial injury that is inherently at greater risk of progression, or alternatively, multiple contusions may coalesce as they progress, resulting in a greater cumulative growth.

Presence of peri-contusional edema in association with the initial contusion may also be indicative of risk of progression. In a small study of 21 patients, Beaumont et al. observed a rim of peri-contusional edema more frequently on the initial scan of contusions that did not later progress compared to those that did [19]. Presence of peri-contusional edema likely represents contusions at a later stage in their natural history which are no longer inclined to progress.

2. Coexisting lesions

The presence of coexisting SDH or SAH on the initial CT scan is predictive of subsequent contusion progression [6, 11, 12, 20, 22, 23]. Presence of SDH increases the risk of progression by two- to threefold, and presence of SAH increases risk by two- to sixfold [6, 11, 12, 22, 23]. In a study by Chang et al., although both SAH and SDH were predictive of any increase in contusion size, only SDH was predictive of progression above a threshold of 5 cm^3^ [12]. Presence of extradural or intraventricular hemorrhages does not appear to be associated with subsequent contusion progression [2, 6, 11, 12].

Concomitant lesions may represent imaging markers of the severity of initial injury, whereby patients who have multiple intracranial pathologies have experienced greater initial trauma than patients with contusion alone. The mechanisms by which concurrent SDH or SAH confers increased risk, while EDH and IVH do not, are unclear. SAH and SDH are associated with local responses including focal ischemia, reperfusion injury, vasogenic edema, and reduced CBF [53], which may provide an additive effect to those responses seen in the traumatic penumbra of the contusion, exacerbating the progression of contusions. SDH may also be secondary to a burst lobe from an underlying contusion, which itself may be more susceptible to progression [2, 54].

3. Additional CT features

Other radiological features from initial CT including basal cistern compression [6, 12, 14, 23], presence of midline shift [7], presence of skull fracture [21, 22], and Marshall Score [12, 16] have been identified as correlating with likelihood of contusion progression. However, of these, only cisternal compression and skull fracture have been shown as independent predictors [14, 21, 23].

4. Contrast extravasation

CT angiography (CTA) is extensively used in the investigation of patients with spontaneous intracerebral hemorrhage (ICH). Measurements obtained from CTA, including the ‘spot sign’ (contrast extravasation [CE]), and its subsequent iterations such as the modified spot sign (leakage sign) and spot sign growth (rate of CE), have been shown to predict ICH expansion, poor outcome, and high mortality among this cohort of patients [55]. Despite the utility of CTA in predicting spontaneous ICH expansion, there have been limited studies on its use in the setting of TBI.

Huang et al. were among the first to investigate the relationship between CE and traumatic contusion growth. In a small cohort of 22 patients, presence of CE was able to predict contusion progression with a sensitivity of 75%, specificity of 78.6%, positive predictive value (PPV) of 66.7%, and negative predictive value (NPV) of 84.6% [56]. Similar findings were subsequently seen in a larger study of 121 patients by Rosa et al., with comparable PPV and NPV at 61.1% and 90%, respectively. In this study, presence of CE was associated with a 14-fold increased risk of contusion progression [57]. Patients showing CE, however, had higher initial contusion volume compared to those not showing CE; as non-contrast CT studies have demonstrated associations between progression and initial volume, this may explain the high predictive value of CE seen in the study by Rosa et al. [57].

Orito et al. examined the use of the leakage sign in traumatic contusion progression in 33 patients; a positive leakage sign was defined as > 10% increase in Hounsfield units between arterial and delayed phase images in the same region of interest. The leakage sign displayed a greater predictive value compared to CE, with a sensitivity of 100%, specificity of 92.8%, PPV of 94.4%, and NPV of 100% [52]. Patients with concomitant SDH, EDH, and diffuse axonal injury were excluded from this study; the predictive ability of the leakage sign may therefore be lower in a true population of TBI patients, where presence of multiple coexistent lesions is typical.

All three studies discussed above used single-energy CT imaging for follow-up after CTA. Bodanapally et al. have demonstrated that in such cases, presence and rate of contusion progression may be overestimated; here, what is interpreted as progression is actually a ‘pseudohaematoma,’ which represents retention of iodinated contrast within the traumatic penumbra following CTA [58]. Since both CE and contusion progression are related to the extent of endothelial damage, degree of overestimation is proportional to the extent of contusion progression. Presence of progression is more accurately determined with the use of dual-energy CT.

Contrast extravasation on gadolinium enhanced MRI may also be potentially useful in the prediction of contusion progression. This method, however, has only been used in one small study of 10 patients; further studies are therefore needed to validate its use in TBI [59].

## Prediction scores

Prediction scores based on admission characteristics have recently been developed and validated for the prediction of progressive hemorrhagic injury [26, 27]. For contusion growth, Allison et al. have proposed a simple four-point predictive score, with an area under the curve of 0.77, comprising three radiologic features: presence of SAH (2 points), presence of SDH (1 point), and presence of skull fracture (1 point) [22].

A more complex prediction model based on a nomogram, developed by Cepeda et al., included additional variables encompassing the clinical background and condition of the patient [14]. These features were: age, initial contusion volume < 5 ml, craniectomy, cisternal compression, hypoxia, and fall as the mechanism of injury [14]. The area under the curve of this model was 0.72, lower than that of the simpler and more accessible model by Allison et al. Neither of these prediction models have been externally validated.

## Decompressive craniectomy

Decompressive craniectomy (DC) is a useful intervention for raised intracranial pressure refractory to medical management, with the subsequent increase in brain compliance contributing to improved cerebral perfusion pressure and reduced risk of brain herniation [60]. The resultant loss in tamponade effect, however, may contribute to contusion progression; progression is seen in 13–58% of patients in the initial few days following DC and, in most cases, occurs in the hemisphere ipsilateral to the surgery [14, 61–63]. Although Cepeda et al. found that patients undergoing DC were three times more likely to develop progression compared to those with no surgical intervention [14, 23], Sturiale et al. did not find any significant difference between these two groups [62].

In a similar manner to medically managed TBI patients, initial contusion volume and presence of SDH on initial CT scan appear to predict risk of contusion progression following DC [64], with an initial contusion volume of greater than 20 ml better predicting subsequent progression than SDH presence [64]. TBI severity, as determined by initial Rotterdam score, is also correlated with both a higher risk, and greater volume, of contusion progression [61, 65]; in a study by Flint et al., a Rotterdam score ≥ 5 represented a 79% chance of progression [61].

## Clinical progression and outcomes

Although associations between contusion progression and neurological outcomes of TBI patients have been extensively reported in the literature, contusion progression may simply represent severe TBI, rather than providing a direct influence on outcome. However, it is also possible that more severe TBI may mediate some of its outcome impact through contusion progression. This distinction is of more than academic interest: If contusion expansion is simply a biomarker of severe injury and poor outcome, interventions to prevent progression are unlikely to reduce the associated morbidity and mortality.

Prediction of contusion progression can nevertheless provide valuable information regarding the patient’s clinical course. Of particular interest is the ability to identify patients who are at risk of progression to a surgical contusion and/or requirement for intubation, ventilation, and neuromonitoring. Benefits of early surgery on 6-month mortality have been seen in such patients, particularly among those presenting with a GCS 9–12 [66]. Contusion progression is typically accompanied by a fall in GCS score during hospital admission [8, 9, 13]; in a study by White et al., each cubic centimeter increase in volume between first and second scan was associated with a 0.2 decrease in GCS [8]. Although clinical deterioration itself is often an indication for surgery, presence of radiological progression alone is also an independent predictor of future requirement for surgical intervention; Chang et al. showed that patients with contusion growth > 5 cm^3^ were seven times more likely to require surgery than those whose contusions grew by < 5 cm^3^ [12]. Patients showing progression also have a longer length of stay in the intensive care unit and in hospital [11, 15, 16], as well as longer ventilatory requirement [15, 16], and higher rates of in-hospital infection [16].

Although some studies report presence of contusion progression and absolute volume increase to be associated with neurological outcome at discharge and up to 12 months later, this has not consistently been observed [14–17]. Juratli et al. showed that a modified Rankin Scale score ≥ 4 was fivefold more likely at discharge and fourfold more likely at 12-month follow-up in patients showing progression compared to those who did not progress [15]. Both the presence and volume of progression were significantly associated with unfavorable outcome at 6 months (Glasgow Outcome Score [GOS] ≤ 3) in a study by Cepeda et al. [23]. Similarly, Qureshi et al. reported higher proportions of patients achieving favorable outcome at 6 months, as measured by both the Extended GOS (GOSE) and Disability Rating Scale score, in patients with non-progressive contusions compared to those that did progress [16]. Iaccarino et al., however, did not find contusion progression to be predictive of unfavorable outcome (GOSE ≤ 4) at 6-month follow-up [13]. In patients undergoing DC, an increase in contusion size of greater than 20 ml postoperatively was associated with a higher likelihood of poor outcome (GOS ≤ 3) and mortality at 6 months [61].

A number of studies have demonstrated univariate associations between contusion progression and short- and long-term mortality [8, 15, 16]. These associations, however, have not been seen in multivariate analyses, indicating that factors other than contusion progression play a more important role in determining the outcome of such patients. Iaccarino et al., for example, have identified neurological deterioration to be a better prognostic indicator compared to contusion progression [13].

## Conclusion

**Fig. 1:**
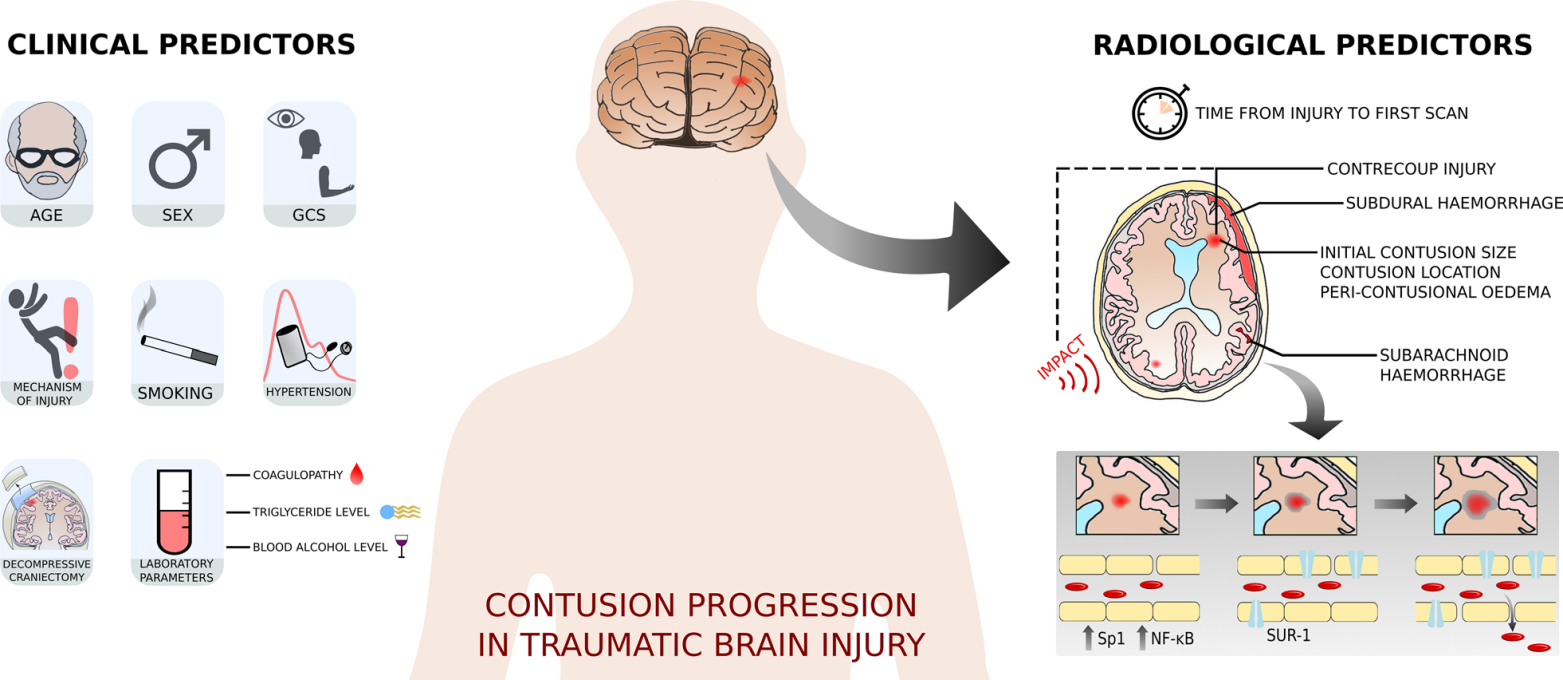
Mechanism of contusion progression and its clinical and radiological predictors. Kinetic energy delivered to mechanosensitive endothelial cells during impact induces upregulation of specificity protein 1 (Sp1) and nuclear factor-ĸB (NF-ĸB) which, in turn, upregulates sulfonylurea receptor 1 (SUR-1). There is a resultant increase in blood–brain barrier permeability and edema formation, followed by capillary fragmentation and extravasation of blood, i.e., contusion progression

Progression of cerebral contusions following TBI is a common cause of neurologic deterioration, may have an additional influence on morbidity and mortality, and is potentially avoidable. The ability to predict which patients with contusions will show progression, before this occurs, could therefore help clinicians better stratify patients and plan clinical management. Clinical factors which may predict contusion progression include initial GCS, history of hypertension, current smoking, coagulopathy, and decompressive craniectomy. Radiological predictors include initial contusion size, contusion location, presence of concurrent SAH or SDH, and absence of peri-contusional edema. A summary the mechanism of contusion progression and clinical and radiological predictors is shown in Fig. 1.
